# Early Detection of Ventilation-Induced Brain Injury Using Magnetic Resonance Spectroscopy and Diffusion Tensor Imaging: An *In Vivo* Study in Preterm Lambs

**DOI:** 10.1371/journal.pone.0095804

**Published:** 2014-04-23

**Authors:** Béatrice Skiöld, Qizhu Wu, Stuart B. Hooper, Peter G. Davis, Richard McIntyre, Mary Tolcos, James Pearson, Ruth Vreys, Gary F. Egan, Samantha K. Barton, Jeanie L. Y. Cheong, Graeme R. Polglase

**Affiliations:** 1 Neonatal Services, The Royal Women's Hospital, Melbourne, Victoria, Australia; 2 Monash Biomedical Imaging, Monash University, Clayton, Victoria, Australia; 3 CSIRO Materials Science and Engineering, Clayton, Victoria, Australia; 4 The Ritchie Centre, Monash Institute of Medical Research, Monash University, Clayton, Victoria, Australia; 5 Department of Obstetrics and Gynaecology, Monash University, Clayton, Victoria, Australia; 6 Victorian Infant Brain Studies, Murdoch Childrens Research Institute, Melbourne, Victoria, Australia; 7 Department of Obstetrics and Gynaecology, University of Melbourne, Melbourne, Victoria, Australia; University of Maryland, College Park, United States of America

## Abstract

**Background and Aim:**

High tidal volume (V_T_) ventilation during resuscitation of preterm lambs results in brain injury evident histologically within hours after birth. We aimed to investigate whether magnetic resonance spectroscopy (MRS) and/or diffusion tensor imaging (DTI) can be used for early *in vivo* detection of ventilation-induced brain injury in preterm lambs.

**Methods:**

Newborn lambs (0.85 gestation) were stabilized with a “protective ventilation” strategy (PROT, n = 7: prophylactic Curosurf, sustained inflation, V_T_ 7 mL/kg, positive end expiratory pressure (PEEP) 5 cmH_2_O) or an initial 15 minutes of “injurious ventilation” (INJ, n = 10: V_T_ 12 mL/kg, no PEEP, late Curosurf) followed by PROT ventilation for the remainder of the experiment. At 1 hour, lambs underwent structural magnetic resonance imaging (Siemens, 3 Tesla). For measures of mean/axial/radial diffusivity (MD, AD, RD) and fractional anisotropy (FA), 30 direction DTI was performed. Regions of interests encompassed the thalamus, internal capsule, periventricular white matter and the cerebellar vermis. MRS was performed using a localized single-voxel (15×15×20 mm^3^, echo time 270 ms) encompassing suptratentorial deep nuclear grey matter and central white matter. Peak-area ratios for lactate (Lac) relative to N-acetylaspartate (NAA), choline (Cho) and creatine (Cr) were calculated. Groups were compared using 2-way RM-ANOVA, Mann-Whitney U-test and Spearman's correlations.

**Results:**

No cerebral injury was seen on structural MR images. Lambs in the INJ group had higher mean FA and lower mean RD in the thalamus compared to PROT lambs, but not in the other regions of interest. Peak-area lactate ratios >1.0 was only seen in INJ lambs. A trend of higher mean peak-area ratios for Lac/Cr and Lac/Cho was seen, which correlated with lower pH in both groups.

**Conclusion:**

Acute changes in brain diffusion measures and metabolite peak-area ratios were observed after injurious ventilation. Early MRS/DTI is able to detect the initiation of ventilation-induced brain injury.

## Introduction

Preterm infants are at high risk of brain injury and long-term neurodevelopmental impairments [1]–[3]. The underlying causes are numerous, with immaturity a key contributor [4] and the immediate newborn period a particularly vulnerable time [5]. Infants born preterm often receive respiratory support at birth, and may be exposed to unintentional injurious ventilation in the delivery room. Schmölzer et al [6] demonstrated that the discrepancy between the estimated tidal volumes (V_T_) during mask-ventilation, and the actual measured V_T_ delivered to the baby, can be substantial, with many infants receiving excessive V_T_. High V_T_ ventilation is harmful both for the lungs [7] and the brain [8]. In preterm lambs, high V_T_ ventilation causes cerebral blood flow instability, white matter injury, inflammation, vascular leakage and oxidative stress, evident as early as 90 minutes after birth. It is not possible to detect brain injury histologically in survivors of preterm birth; instead, magnetic resonance imaging (MRI) provides a potential biomarker for acute brain injury in preterm newborns [9]–[12]. To date, *in vivo* MRI examinations in preterm animals are scarce, complicating translation of the findings to the human setting.

Diffusion tensor imaging (DTI) is an MRI method based on measures of the motion of water molecules in brain tissue and provides information on brain organization and microstructure [13]. The mean displacement of the water molecules is Mean Diffusivity (MD), and Fractional Anisotropy (FA) indexes their degree of preferred diffusion directionality. FA can take values from zero (isotropic; i.e. diffusion is equal in all directions) to one (purely anisotropic; i.e. entirely in one direction) and has been shown to increase with age and tissue maturity, while MD decreases [14]. Other common diffusion measures are Axial Diffusivity (AD) and Radial Diffusivity (RD) describing the magnitude of water diffusion parallel or perpendicular to white matter (WM) tracts, and thought to reflect axonal and myelin integrity respectively [15]. For example, reduced AD and unchanged RD may be seen in acute axonal damage where there is axonal degeneration with comparatively preserved myelin [16]. In contrast, increased RD and unchanged AD is present in animal models of demyelination [17]. In term newborns with hypoxic ischemic encephalopathy (HIE), both axial and radial diffusivity are reduced during the sub-acute phase in infants with poor outcome [18].

Magnetic resonance spectroscopy (MRS) measures brain metabolites and provides *in vivo* information on brain metabolism. Similar to diffusion measures, the neurochemical profile changes during development [19] approaching adult values by around 3 years of age. The common metabolites examined in the newborn brain include lactate, choline, N-acetyl aspartate (NAA) and creatine [12], [20]. Lactate is a marker for anaerobic metabolism, and can be detected in the brain after conditions such as hypoxic ischemic injury [12], [21] and acute neonatal stroke [22]. In the preterm brain, some lactate may be present [23], but levels fall with increased postmenstrual age. Choline-containing compounds are intermediates in phospholipid metabolism and are considered as markers of cell membrane turnover. Choline levels are high early in development, steadily decreasing during the first years of life, whereas increased levels can be seen for example in demyelinating diseases [24]. NAA is present in axons and often considered a marker for neurons, and has been reported to be decreased in acute brain injury [12], [21], [22]. Creatine is a marker for high-energy products such as ATP and important for energy supply in the cells. Creatine and NAA are both low in the preterm brain, with a marked increase leading up to term age [9].

The overall aim of the present study was to investigate whether structural MRI, DTI and MRS can be used for early *in vivo* detection of ventilation-induced brain injury in preterm lambs. We hypothesized that lambs that were stabilized with an “injurious” ventilation strategy at birth would display altered metabolite patterns on spectroscopy, such as increased lactate peak-area ratios, possibly in association with decreased creatine and NAA and increased choline ratios. Moreover, we hypothesized that high V_T_ ventilation would have adverse effects on brain microstructure and that specific changes in MD/FA/AD/RD would provide information on particularly vulnerable regions and tissue components.

## Methods

### Ethics statement

The experimental protocol was performed in accordance with guidelines established by the National Health and Medical Research Council of Australia and was approved by the Monash Medical Centre animal ethics committee (MMCA) at Monash University (AEC number: MMCA-2012-26).

### Preterm delivery and stabilization

Pregnant anaesthetized ewes underwent caesarean section at 0.85 of gestation (day 125–127; term is 148 d). Lambs were delivered and randomly assigned to receive stabilization with either a “protective ventilation” strategy (PROT, n = 7) or an initial 15 minutes of “injurious ventilation” (INJ, n = 10) as described previously [8]. Briefly, the PROT strategy consisted of intubation, administration of intratracheal surfactant (Curosurf 100 mg/kg) and one sustained inflation for 30 seconds at peak inflation pressure (PIP) 35 cmH_2_O delivered with a Neopuff (Fisher & Paykel Healthcare, Panmure, Auckland, New Zealand). Ventilation (Babylog 8000+; Dräger, Lübeck, Germany) was commenced 1 minute prior to clamping of the umbilical cord, using volume guarantee (VG) mode with a set V_T_ of 7 mL/kg and a positive end expiratory pressure (PEEP) of 5 cm H_2_O (inspiratory time 0.3 sec, expiratory time 0.6 sec). The INJ strategy consisted of intubation and early cord clamping, followed by VG ventilation with a target V_T_ of 12 mL/kg, no PEEP and administration of Curosurf at 15 minutes. Fractional inspired oxygen (FiO_2_) was initially set at 0.4 in both groups, and then adjusted to maintain arterial oxygen saturation (SaO_2_) between 88–95%. After the initial 15 minutes of intervention, both groups were managed using the PROT strategy for the remainder of the experiment.

### Monitoring and care

All lambs were dried, placed on a heated mattress, and fitted with rectal temperature and transcutaneous arterial oxygen saturation (SpO_2_) probes (Masimo, CA USA). Umbilical catheters were placed for intravenous administration of anesthesia, arterial blood gas sampling and invasive real-time monitoring of heart rate (HR) and blood pressure (BP) (Powerlab: ADInstruments, Castlehill, NSW, Australia). Ventilator settings were noted regularly. The lambs were anaesthetized throughout the study using a continuous infusion of Alfaxane (Jurox, East Tamaki, Auckland, New Zealand) 10 mg/kg/h in 5% dextrose. Arterial blood samples were collected regularly and measured for partial pressure of oxygen (PaO_2_), partial pressure of carbon dioxide (PaCO_2_), pH, Base Excess and lactate (ABL30, Radiometer, Copenhagen, Denmark). The ewes were humanely euthanized using sodium pentobarbitone (100 mg/kg i.v) after the caesarean section, and the lambs were euthanized after completion of the MRI.

### Magnetic resonance imaging

After 60 minutes of ventilation, the lambs were moved from the stabilization area to the MR scanner, using the BabyPAC portable and MR compatible ventilator (Pneupac, Smiths Medical, UK) in controlled mandatory ventilation mode. Lambs were imaged in supine position, swaddled and monitored using continuous HR and SpO_2_ monitoring and blood-gas analyses every 15 minutes.

All scans were performed on a 3T MR scanner (Siemens Skyra, Erlangen) at Monash University Biomedical Imaging (Clayton, VIC, Australia), using a 15-channel RF coil for both RF transmission and receive. The MRI protocol consisted of structural imaging sequences (T1, T2 and DTI), susceptibility weighted imaging (SWI) and a single voxel MRS. The total scanning time was approximately 40 minutes.

A 3D MPRAGE sequence was used to acquire T1-weighted images with parameters: repetition time (TR)/echo time (TE)/inversion time (TI) = 1440/3.92/900 ms, flip angle = 9°, field of view (FOV) = 200×200 mm^2^, slab thickness  = 102.5 mm, data matrix  = 256×256×128, resulting in a voxel size  = 0.78×0.78×0.8 mm^3^, number of averages  = 2. T2-weighted images were acquired with a 3 D SPACE sequence with parameters: TR/TE  = 1000/132 ms, flip angle  = 120°, FOV = 192×192 mm^2^, 44 mm slab thickness, acquisition matrix  = 384×384×88, voxel size  = 0.5×0.5×0.5 mm^3^, number of averages  = 2. SWI was acquired using a standard 3 D gradient echo sequence, with TR/TE  = 28/20 ms, flip angle  = 15°, FOV = 176×132 mm^2^ and 72 mm slab thickness, acquisition matrix  = 288×216×60, voxel size  = 0.6×0.6×1.2 mm^3^. The second phase encoding direction (in the 1.2 mm voxel direction) was orientated parallel to the main magnetic field to produce the best susceptibility effect.

DTI was acquired in the axial plane using a spin-echo EPI sequence with parameters: TR/TE  = 11400/99 ms, FOV  = 154×154 mm^2^, data matrix  = 128×128, 50 continuous slices with 1.2 mm thickness, voxel size  = 1.2×1.2×1.2 mm^3^. Diffusion encoding gradients were applied in 30 directions with b value  = 1500 s/mm^2^. The diffusion-weighted acquisition was repeated twice and 5 b = 0 volumes were acquired.

**Figure 1:**
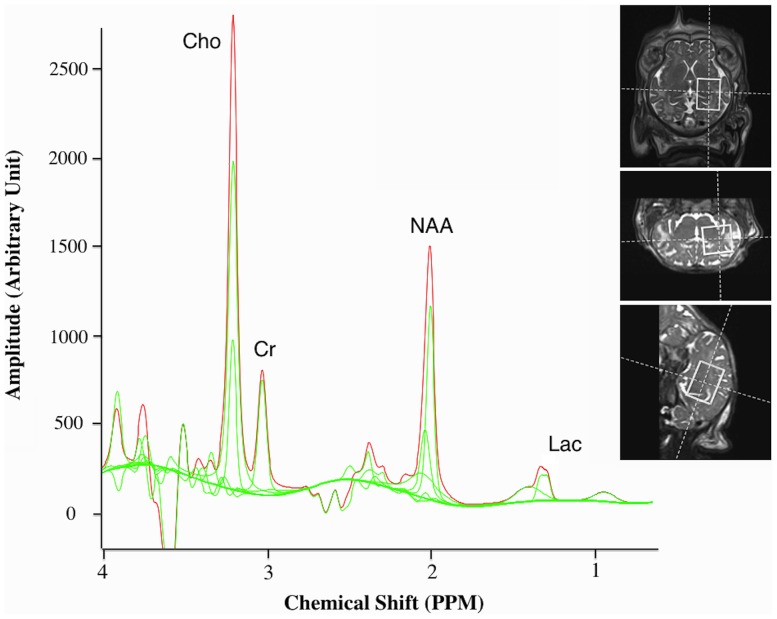
Magnetic resonance spectroscopy. Sample 270(TE) spectra from one lamb that received injurious ventilation with a representative example of voxel placement (right). Cho; choline, Cr; creatine, NAA; N-acetylaspartate, Lac; lactate. Peak ratios were determined for Lac/Cr, Lac/NAA, Lac/Cho, NAA/Cr, NAA/Cho and Cho/Cr.

MRS was performed using a localized single-voxel spin echo sequence with TR/TE  = 2000/270 ms and voxel size  = 15×15×20 mm^3^, encompassing supratentorial deep grey matter (GM) and central WM. A representative example of voxel placement and one sample spectra from a single subject are shown in Figure 1.

### MRI data analysis

The structural MR images (T1, T2) and SWI were reviewed for malformations and cerebral injuries such as haemorrhages and infarcts.

**Figure 2:**
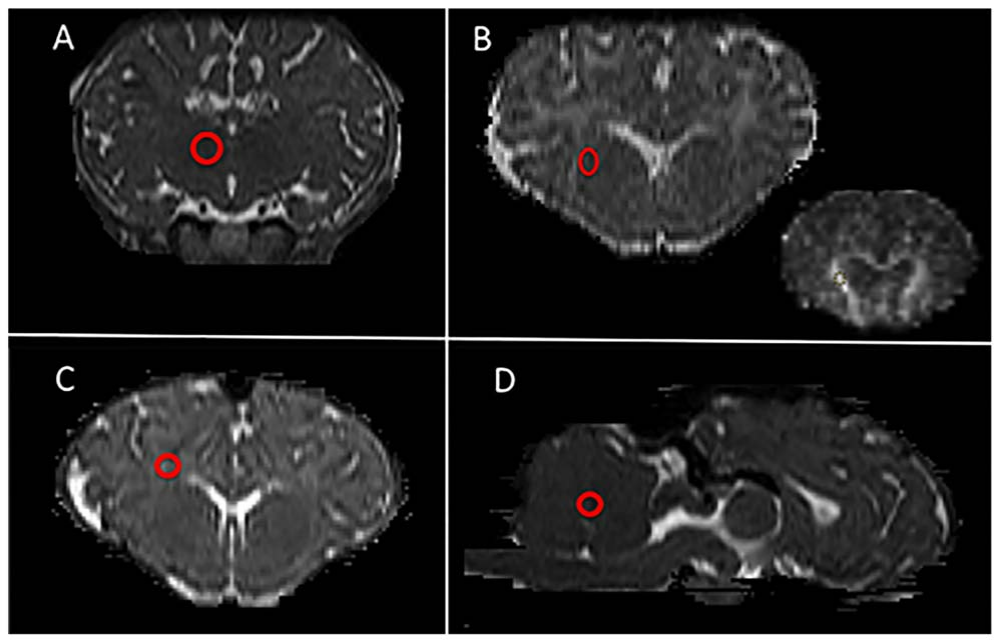
Regions of interest placement for DTI analyses. Representative example of ROI placements (red circles) on high-resolution T2 images (A-C, coronal orientation; D, sagittal orientation). A: thalamus, B: internal capsule (inset: corresponding MD map), C: periventricular white matter, D: cerebellar vermis

DTI data was processed using the FMRIB Software Library (FSL, FMRIB, Oxford, UK [25]). Images were first converted to NIFTI format then a brain mask was generated for each lamb by manual drawing. After eddy-current correction, FA, MD and other parametric maps were generated using the FMRIB's Diffusion Toolbox (FDT). The first eigenvalue was used as the AD and the second and the third eigenvalues were averaged as the RD. All maps were then coregistered into the lamb's high-resolution T2 image using Linear Image Registration Tool (FLIRT [26]) using the b0 image as a reference. Regions of interest (ROIs) were drawn on high-resolution T2 images and then exported to the FA, MD, AD and RD maps to extract regional values. The mean value from three adjacent slices was calculated for each of the four ROIs. The Sheep Brain Atlas from Michigan State University (Brain Biodiversity Bank, National Science Foundation) was used for accurate identification of the thalamus, internal capsule, periventricular WM and the cerebellar vermis aiming for the midline WM. A representative example of ROI placements is shown in Figure 2.

MRS data was processed using the software TARQUIN which implements spectral fitting in time domain [27]. Peak-area ratios for lactate (Lac) relative to NAA (sum of N-acetylaspartate and N-acetylaspartylglutamate), choline (Cho, sum of glycerophosphocholine and phosphocholine) and creatine (Cr, sum of free creatine and phosphocreatine) were calculated, as well as peak-area ratios for NAA/Cr, NAA/Cho and Cho/Cr.

### Statistical analysis

The study sample was divided into those stabilized with the PROT strategy and those with the INJ strategy and groups were compared using Student's t-test, Mann-Whitney U-test and 2-way repeated measures-ANOVA with a Holm Sidak Post hoc test. Spearman's correlations were used to investigate correlations between MRS peak-area ratios, diffusion parameters and pH.

Data are presented as mean (± standard deviation, SD) and range. A p-value of <0.05 was accepted as significant.

## Results

### Baseline characteristics and physiological parameters

The mean birth weight of the study sample was 3250 (±245) grams (range 2800–3800 g), with no significant difference between the groups: PROT 3329±250 g versus (vs) INJ 3189±237 g. The mean gestational age was 125.8±1 days (range 125–127 d), and similar between groups. There were 8 female and 9 male lambs, and these were similarly distributed between groups. Heart rate, peripheral oxygen saturation (SpO_2_), systolic, diastolic and mean BP were similar in both groups at all investigated times during and after the intervention (data not shown).

### Ventilations parameters

**Figure 3:**
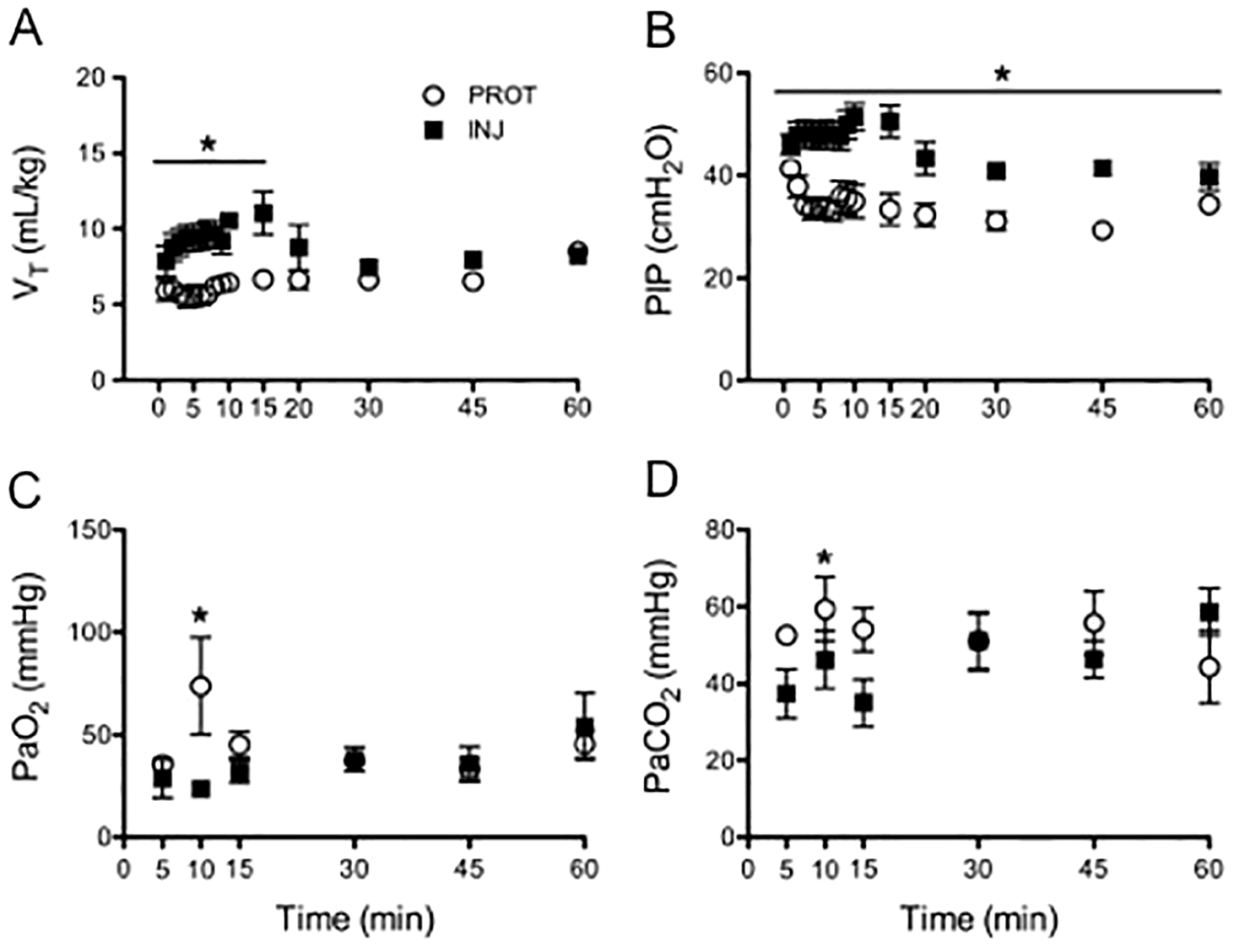
Ventilation and oxygenation. Ventilation and arterial blood gas measurements in lambs ventilated with an injurious strategy (black squares) versus a protective strategy (open circles) during the first 15 minutes, followed by maintenance ventilation until 1 hour. A) Tidal volume (V_T_; Group: p = 0.002, F = 19.14; Time: p = 0.58, F = 0.87; Group × Time: p = 0.17, F = 1.42) B) Peak inspiratory pressure (PIP: Group: p = 0.002, F = 19.24; Time: p<0.001, F = 5.72; Group × Time: p = 0.07, F = 1.70) C partial pressure of arterial (Pa) oxygen (PaO_2_; Group: p = 0.87, F = 0.03; Time: p = 0.13, F = 1.6; Group × Time: p = 0.03, F = 2.2) and D) Pa carbon dioxide (PaCO_2_; Group: p = 0.55, F = 0.37; Time: p = 0.49, F = 0.88; Group × Time: p<0.001, F = 5.21)

Injuriously ventilated lambs had higher V_T_/kg (p = 0.002) and lower PEEP (p<0.001) during the first 15 min compared to PROT lambs as expected. The PIP was higher throughout the study in INJ lambs (p = 0.002; Figure 3). The initial FiO_2_ was 0.4 in both groups, with a significant increased oxygen requirement in the INJ group compared to the PROT group (p = 0.04) from 15 min.

### Arterial blood gas parameters

Lambs in the INJ group had lower PaO_2_ at 10 min (INJ: 22±7 vs PROT: 52±49 mmHg, p = 0.01), and lower PaCO_2_ at 15 min (INJ: 35±19 vs PROT: 54±15 mmHg, p = 0.04), compared to the PROT group (Figure 3). Consequently, at 15 min, the INJ lambs had a higher pH (INJ: 7.5±0.2 vs PROT: 7.2±0.1, p = 0.006). At experimental endpoint however, INJ lambs were more acidotic compared to lambs in the PROT group (INJ: mean pH 7.09±0.2 vs PROT: 7.28±0.2, p = 0.03).

### Magnetic resonance and diffusion tensor imaging

**Figure 4:**
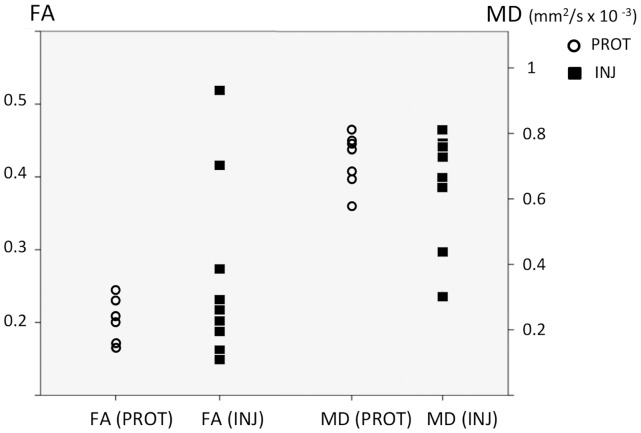
Diffusion measures in the cerebellum. Individual values of fractional anisotropy (FA) and mean diffusitivity (MD) within the cerebellum of protectively ventilated (PROT; open circles) and injuriously ventilated (INJ; black squares) preterm lambs, measured in 30 directions using diffusion tensor imaging. Two of the injuriously venilated lambs had higher FA and lower MD (p<0.05) in the cerebellum compared to the other lambs.

Inspection of conventional MR images revealed no gross cerebral injury or abnormalities in either group. Diffusion data was successfully obtained in all 7 PROT lambs and in 9 of the 10 INJ lambs. In thalamus, the INJ lambs had higher mean FA (INJ: 0.20±0.02 vs PROT: 0.18±0.02, p = 0.049) and lower mean RD [(7.8±0.3)×10^−4^ mm^2^/sec vs (8.4±0.3)×10^−4^ mm^2^/sec, p = 0.002] compared to PROT lambs, whereas AD and MD were not significantly different. Two of the INJ lambs had significantly higher FA, lower MD, lower AD and lower RD (p<0.05 for all) in the cerebellum compared to the other animals (Figure 4), but no group level diffusion differences were seen in the cerebellum when comparing the INJ to the PROT lambs.

Mean arterial pH correlated negatively with FA (p = 0.013, Spearman's rho = −0.56) and AD (p = 0.010, Spearman's rho = −0.58) in the internal capsule but not in the other investigated ROIs.

### Peak-area MRS ratios

**Figure 5:**
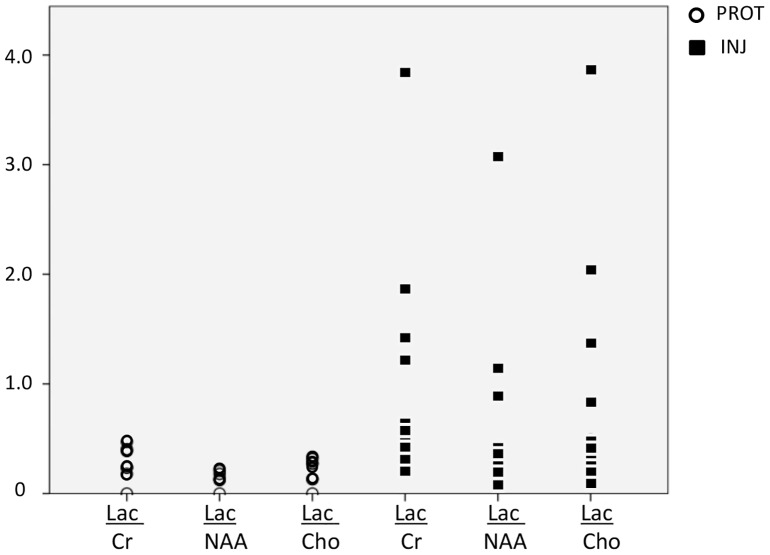
Peak-area MRS lactate ratios. Individual peak area MRS ratios using a single voxel encompassing supratentorial deep grey matter and central WM in protectively ventilated (PROT; open circles) and injuriously ventilated (INJ; black squares) preterm lambs. LAC; lactate, Cr; creatine, NAA; N-acetylaspartate, Cho; choline. Compared to the PROT group, MRS peak-area ratios of lactate to other metabolites (Cr, NAA, Cho) were more scattered in INJ lambs.

Compared to the PROT group, MRS peak-area ratios of lactate relative to other metabolites (Cr, NAA, Cho) were more scattered in INJ lambs (Figure 5). At a group level, a trend for higher mean ratios for Lac/Cr (INJ: 0.92±1.1 vs PROT: 0.27±0.2, p = 0.051) and Lac/Cho (INJ: 0.81±1.1 vs PROT: 0.21±0.1, p = 0.056) was observed, which correlated with lower pH in both groups (INJ: p = 0.043, Spearman's rho = −0.496, and PROT: p = 0.045, Spearman's rho = −0.492). At an individual level, three of the 10 INJ lambs had significantly higher Lac/Cr and Lac/Cho ratios compared to the other INJ lambs and in comparison to all other animals: Lac/Cr: 2.15±1.5 vs INJ: 0.39±0.2 and 2.15±1.5 vs PROT: 0.33±0.2, p<0.05, Lac/Cho: 2.02±1.6 vs INJ: 0.29±0.1 and 2.02±1.6 vs PROT: 0.25±0.1, p<0.05 (Figure 5). Peak-area ratios for NAA/Cr, NAA/Cho and Cho/Cr were not different between INJ and PROT lambs.

## Discussion

High V_T_ ventilation during the initial stabilization of preterm lambs causes white matter gliosis, inflammation and vascular leakage compared to lambs that received a protective ventilation strategy [8]. These pathologies are evident within 90 minutes after birth and are likely to contribute to permanent brain injury. In the present study, we investigated whether *in vivo* imaging, using advanced MRI techniques, could detect ventilation-induced brain injury. While no macroscopic injury was observed using conventional MRI, we observed acute changes in diffusion measures and metabolite peak-area ratios in injuriously ventilated lambs compared with protective ventilated lambs, which may reflect the initiation of brain injury.

In analogy with our histopathological study [8], no gross injuries such as hemorrhages or infarcts were seen on structural MRI after high V_T_ ventilation. This was an expected finding supported by longitudinal studies where acute injury, although originating in the immediate newborn period, evolves over time and is not evident until many hours, or even days after birth [21]. Our previous evidence of inflammation and injury at the microstructural level [8], that could not be detected using structural MRI in the current study, highlights the need for alternative MR sequences like DTI and MRS, as well as the importance of the imaging time point post insult.

In a recent large study of very preterm neonates, Card et al [28] performed MRS during the first 2 weeks after birth and demonstrated that elevated peak-area ratios for Lac/Cr and Lac/Cho were significantly associated with severity of illness scores at birth (Apgar score at 1 and 5 minutes and Clinical Risk Index for Babies II). These findings are in agreement with the present results where increased peak-area ratios for Lac/Cr and Lac/Cho were observed in INJ lambs in association with a low arterial pH, suggesting early compromise. However, in both studies large inter-subject variations were observed; in preterm humans it may reflect the different timing of the “insult” where as in our experimental model, the wide variation may have been partially explained by the smaller sample size. In relation to outcome however, several studies have shown that elevated lactate peak-area ratios indicate a poor prognosis [12], [18], [29], [30].

We were unable to demonstrate differences in peak-area ratios for NAA/Cr, NAA/Cho or Cho/Cr between intervention groups. As these ratios are thought to indicate neuronal integrity and cell membrane turnover [31], [32], the early time point after intervention at which MR imaging was performed may explain why this was not evident in the present study. For example, in the study by Card et al [28], early NAA/Cr was associated with white matter injury on conventional MRI at term equivalent age, but not with birth severity of illness scores. In a similar study of very preterm infants scanned around 2–3 weeks of age [33], the peak-area ratios of Lac/Cho were not elevated, whereas lower NAA/Cho was observed in infants with white matter injury when scanned again at term age. However, reports are conflicting; Duerden et al [34] found lower NAA/Cho peak-area ratios (but no change in Lac/Cho) at 2–3 weeks of age in very preterm infants who received intense resuscitation at birth compared with those requiring less intervention. In term infants with HIE, and thus perhaps more acute patterns of injury than in the preterm population, both early [12], [35] and later [18], [36] spectroscopy studies have demonstrated that low levels of NAA and Cho are accurate prognostic markers of poor neurodevelopmental outcome, and evident even after hypothermia treatment [18]. Additional spectroscopy data is needed to provide information on metabolite patterns in the immediate newborn period.

Several studies in neonates have used DTI to investigate neurodevelopment and injury [37]–[39]. In the white matter, MD decreases and FA increases with increasing postnatal age, reflecting maturation of fiber tracts and myelination processes [40]. In contrast, the cortical grey matter shows increasing MD and decreasing FA over time, due to a decreasing radial organization [41]. In term and near term infants with HIE, DTI values are generally characterized by a decreased MD within the first 24 hours, reaching minimum values around day 3 followed by a so called pseudonormalization around day 7 [42]. FA values decrease over the first days and remain low with no sign of pseudonormalization [43]. The majority of studies show that these early diffusion changes are correlated with poor outcome [18], [29]. Our findings of significantly decreased MD, AD and RD in the cerebellum in two of the nine INJ lambs compare favorably with other DTI studies suggesting local tissue distress [44], [45]. However, the associated increased FA was an unexpected finding. Similarly, the mean FA was higher in thalamus in the INJ group, while RD was lower compared to PROT lambs. We speculate that this may relate to the early time-point at which imaging occurred post-ventilation in the present study (i.e. 60–90 minutes). In an adult rat model of traumatic brain injury, where DTI was performed 2 hours after the insult, increased FA along with decreased MD, AD, and RD were found in several brain regions both adjacent and distant to the injury site, interpreted by the authors to be consistent with cytotoxic edema [46]. Indeed, timing of imaging is essential, and additional research is warranted to further delineate the temporal evolution of diffusion alterations in the compromised preterm brain.

The current animal model provides several translational advantages. The sheep brain has many similarities with the human in terms of anatomy, physiology and development [47]. At 126 days of gestation, the lamb brain corresponds approximately to that of a 34–36 week infant and is very vulnerable to white matter injury [8], [48]. Although infants born extremely preterm are at highest risk of severe impairment, the considerably larger group of moderately preterm births implies a greater overall health problem [49]. Accordingly, this group of infants is a prioritized target for neuroprotective strategies [50]. The present study adds to our understanding of neonatal brain injury in this important patient category; it validates the use of combined structural MRI, DTI and MRS, as well as the assessment of multiple regions. Yet, the foremost strength of the present study is the *in vivo* imaging approach that to our knowledge has not been conducted previously in ventilated lambs.

A limitation of the present study is that the lamb lungs at 0.85 of gestation are functionally equivalent to a 25–27 week preterm infant [51], and are thus more immature than the brain, therefore results should be interpreted with caution. For the diffusion analyses, although aiming for anatomical accuracy, it is possible that the chosen ROIs were heterogeneous with regards to tissue content. Moreover, it would have been valuable to analyze MRS in the cerebellum where we observed altered diffusion, unfortunately this was technically difficult to perform. An investigation of the histological correlates of the findings will strengthen the interpretation of the results, and is ongoing.

In conclusion, injurious ventilation during stabilization of preterm lambs has early adverse effects on the brain that are detectable with MRS and DTI. The observed changes may precede morphological abnormalities that are not seen on conventional MRI and could reflect progression towards overt and/or permanent brain injury. Further *in vivo* assessment of brain metabolites and diffusion parameters may provide useful indicators of effective neuroprotective strategies. Importantly, the present research contributes to bridging the gap between neonatal cardio-pulmonary and brain imaging research findings.
